# A low-cost repellent for malaria vectors in the Americas: results of two field trials in Guatemala and Peru

**DOI:** 10.1186/1475-2875-6-101

**Published:** 2007-08-01

**Authors:** Sarah J Moore, Samuel T Darling, Moisés Sihuincha, Norma Padilla, Gregor J Devine

**Affiliations:** 1 Ifakara Health Research and Development Center, Ifakara, Tanzania; 2 Puerta del Cielo Foundation, Canada/ Guatemala; 3 Ministerio de Salud, Iquitos, Peru; 4 Universidad del Valle, Guatemala City, Guatemala; 5 Rothamsted Research, Harpenden, UK

## Abstract

**Background:**

The cost of mosquito repellents in Latin America has discouraged their wider use among the poor. To address this problem, a low-cost repellent was developed that reduces the level of expensive repellent actives by combining them with inexpensive fixatives that appear to slow repellent evaporation. The chosen actives were a mixture of para-menthane-diol (PMD) and lemongrass oil (LG).

**Methods:**

To test the efficacy of the repellent, field trials were staged in Guatemala and Peru. Repellent efficacy was determined by human-landing catches on volunteers who wore the experimental repellents, control, or 15% DEET. The studies were conducted using a balanced Latin Square design with volunteers, treatments, and locations rotated each night.

**Results:**

In Guatemala, collections were performed for two hours, commencing three hours after repellent application. The repellent provided >98% protection for five hours after application, with a biting pressure of >100 landings per person/hour. The 15% DEET control provided lower protection at 92% (*p *< 0.0001). In Peru, collections were performed for four hours, commencing two hours after repellent application. The PMD/LG repellent provided 95% protection for six hours after application with a biting pressure of >46 landings per person/hour. The 20% DEET control provided significantly lower protection at 64% (*p *< 0.0001).

**Conclusion:**

In both locations, the PMD/LG repellent provided excellent protection up to six hours after application against a wide range of disease vectors including *Anopheles darlingi*. The addition of fixatives to the repellent extended its longevity while enhancing efficacy and significantly reducing its cost to malaria-endemic communities.

## Background

In 2005, the WHO reported that 41% of malaria cases occur outside of Africa [1]. This marked a significant increase over their 2001 estimate of 13.6% [2] and reflects a growing awareness of the malaria problem beyond Africa. Greater recognition of this should encourage increased research on malaria vector control in other areas of the globe, an important consideration, as vectors in these regions generally exhibit behavior patterns that make them less susceptible to control measures shown to be effective in Africa, such as insecticide treated bednets (ITNs) and indoor residual spraying (IRS).

These behaviors include tendencies to [i] outdoor resting e.g. *Anopheles darlingi *[3] and *Anopheles dirus *[4]; ii) outdoor feeding e.g. *Anopheles minimus *[4], *An. darlingi*[3], and *Anopheles sinensis *[4]; and iii) significant feeding activity during early evening e.g. *Anopheles albimanus *[5], *Anopheles nuneztovari *[5], *Anopheles farauti *No.2 [6] and *An. darlingi *[7]. A study for implementing ITNs in four Latin America countries showed that 25% of *An. albimanus *in Nicaragua, 28% of *Anopheles punctimacula *in Ecuador, 57% of *An. albimanus *in Peru, and 30% of *An. nuneztovari*, also in Peru, fed before 9:00 pm, when people are still active and often still outdoors [8]. More recently, a case-control study in Colombia [9] showed that ITNs provided only 50% reduction in malaria. The authors attributed this to bites received when people were not sleeping under their nets. Even in areas where ITNs are considered to be highly effective malaria control tools, it appears that their introduction may have caused behavioral shifts among malaria vectors, with outdoor and early evening feeding becoming more frequent among them.

In such conditions, ITNs may be usefully supplemented by an effective insect repellent [10,11]. A recent household randomized trial in Pakistan [12] confirmed that the widespread provision of a repellent soap incorporating DEET and permethrin can reduce the risk of malaria by >50%. Moreover, an unpublished clinical trial in the Bolivian Amazon [13], with a 30% para-menthane-diol (PMD) formulation, showed an 80% reduction in *P. vivax *in those using repellent and ITNs, compared to an ITN only group.

In countries burdened with malaria, nearly all families affected by the disease face severe economic pressure from loss of income and treatment costs. In fact, for the poorest families in Latin America these indirect costs may represent as much as 20% of annual household income [14,15]. In response to these conditions, a low-cost repellent lotion (LCR) was developed by S.T. Darling for distribution to malaria-endemic communities in the Americas.

This plant-based formulation (patent pending) incorporates two principal active ingredients: para-menthane-diol (PMD) and lemongrass oil (LG). PMD is derived from pine extractives or from lemon eucalyptus (*Corymbia citriodora*), a tree that is grown commercially in Latin America and China. PMD is a highly effective, broad-spectrum insect repellent [16-18] that has proven to lower malaria incidence where malaria transmission is maintained by *An. darlingi *[13]. Lemongrass oil, a distillate of *Cymbopogon citratus *or *Cymbopogon flexuosus *leaves, is traditionally used in many parts of the Americas to repel mosquitoes [19] and it is repellent to *An. darlingi *and other disease vectors (Moore *et al*., in preparation). To lower the cost of the repellent and maintain its efficacy, the PMD and LG actives were combined with some low-cost ingredients (fixatives) that extend the repellent effect by slowing the evaporation of volatile repellent actives [20]. The studies reported here were designed to measure whether such a repellent, containing lower amounts of actives than reported in previous studies, could prove its efficacy against malaria vectors and other mosquitoes at a much-reduced cost per application.

## Methods

### Study Site A

The field test was conducted near the Port of Champerico (091°55'W,14°18'N) on the Pacific coast of Guatemala, in June 2005. The estuaries and mangrove swamps that characterize the area are fed by rivers that descend from nearby volcanoes around Quezaltenango. The tests were conducted on a local *finca *– a farm where cattle are raised. The *finca *has a large permanent lagoon and swamp area that is a known *An. albimanus *breeding site. As *An. albimanus *feeds preferentially on cattle [21] it was ensured that livestock were enclosed 50 meters behind the volunteers, so that host-seeking mosquitoes emerging from the breeding site and attracted by the animal odours, would first encounter the collectors.

### Study site B

The trial took place outside of Zungarococha, a small village near Iquitos, the capital of Loreto, Peru (3.8° S 73.2° W). It was staged in late February 2006 in order to coincide with high *An. darlingi *populations, but while malaria transmission remained low. The region is lowland tropical forest with two tributaries of the Amazon River flowing through: the Nanay to the north, and the Itaya to the south. The volunteers collected mosquitoes in a sparsely wooded area that was situated between a group of houses and a lake, or *cocha*, fed by the river that is a permanent breeding site for *An. darlingi*.

### Test Repellents. Study A

The following repellent formulations (% by volume) were used: (1) C15 containing 15% PMD (derived by acid modification of *Corymbia citriodora*; CAS: 42822-86-6; Chemian Technology Ltd), with LG (distilled from *Cymbopogon citratus*; CAS: 8007-02-1 The Essential Oil Company Ltd.), filler and fixative; (2) T15: 15% PMD (derived by acid modification of Citronellal and recovery with aromatic hydrocarbons; CAS 42822-86-6; Takasago International Corporation), with LG, filler and fixative (patent pending); (3) T20: 20% PMD (Takasago International Corporation), with LG, filler and fixative; (4) positive control: 15% DEET (N, N-diethyl-meta-toluamide, CAS 134-62-3; Sigma Aldridge) in ethanol; (5) negative control: filler mix.

### Test Repellents. Study B

Slightly modified repellent formulations (% by weight) were used: (1) PMD/LG containing 16% PMD (Takasago International Corporation), with LG (Berje), filler and fixative (patent pending); (2) positive control: 20% DEET (Sigma Aldridge) in ethanol (Sigma Aldridge); (3) negative control: 20% mineral oil (ExxonMobil Corporation) in ethanol.

### Test Procedures. Study A and B

Both studies were controlled, double-blinded, Latin-square designs that utilized human-landing catches from treated or untreated volunteers in order to measure repellency. All solutions were placed in unmarked containers labelled by code. On any one night, human volunteers had both lower legs treated with either the PMD/LG candidate repellents or a positive or negative control at a rate of 0.002 ml/cm^2 ^between the ankle and the knee. Volunteers' leg length and circumference were measured to calculate surface area, and the correct dose of treatment was measured using a micropipette. Repellent was then applied using a latex glove to minimize absorption of material by the hand of the volunteer. During the human-landing catches, the volunteers wore shorts to the knee, work boots, and a loose bug jacket (ProBuy) to ensure that blood-seeking mosquitoes had access only to their lower legs. After midday, volunteers did not smoke, consume alcohol, or use soap when washing. This was intended to minimize variation in their headspace kairomones [22,23].

The designated locations within the field sites were 10 m from each other and a minimum of 20 m from alternate sources of kairomones such as houses and livestock. As insect repellents act over a distance of less than a meter, and the maximum distance of host attraction of a single human to mosquitoes is 10 m [24], this design minimizes the "relativity effect" wherein insects must choose between two hosts simultaneously. Mosquitoes were collected as soon as they landed on the exposed lower legs of the volunteers, but before probing of the skin commenced, using a mouth aspirator, flashlight, and collection vessel designed for this purpose. Collection vessels were changed each hour to provide hourly measures of repellence. Umbrellas were also provided to protect the volunteers from any rain showers that might wash away their repellent.

### Test Procedures. Study A

The three repellents and two controls were applied to the five volunteers at 14.30 h, and human-landing catches were performed at the field site for one hour before and one hour after sunset (1730–1930 h), when the evening mosquito biting is at its peak as shown by preliminary human-landing catches. The times chosen allowed an assessment of the protection afforded by the repellent over five hours while exposing volunteers to bites for only two hours. This helped minimize exposure and risk to the collectors. The study was a balanced 5 × 5 Latin-square design that required each volunteer to test each treatment five times over a period of 25 nights. Every evening, each individual was allocated one of five treatments, and sat in one of five allocated positions. Consequently, the volunteers changed position every five days.

### Test Procedures. Study B

The repellent and two controls were applied at 16.00 h, and man-landing collections were performed in the field, between 18.00 and 22.00 h, as this is the time of peak *An. darlingi *activity in the area. The times chosen allowed an assessment of the protection afforded by the repellent to be made for a period of six hours while exposing volunteers to bites for only four hours. This helped minimize exposure and risk to the collectors. The volunteers took a 15-minute break between 20.00 and 20.15 h. The study was a 3 × 3 balanced Latin-square design that required each volunteer to test each treatment three times over a period of nine nights, with volunteers changing positions every third night. Due to unforeseen circumstances, one of the volunteers was replaced after four nights, and this was factored into the statistical analysis.

### Ethical Issues

All volunteers were experienced at conducting man-landing catches. A form outlining procedure was given to the volunteers to ensure that they had full understanding of the potential risks of a study of this kind. In addition, each was given a chloroquine (Guatemala) or mefloquine (Peru) prophylaxis in accordance with WHO guidelines. Full ethical approval was obtained from Universidad del Valle, Guatemala (Study A); and from London School of Hygiene and Tropical Medicine Ethics Board and Instituto Nacional de Salud, Peru (Study B).

### Statistical Analysis

Mosquitoes were maintained overnight and killed by cooling prior to identification the following morning. Data were normalized after transformation with natural log (x+1), verified by Anderson-Darling Normality Test, and were analysed with General Linear Model (GLM) using SPSS 13 for Windows. The model measured the effect of position, individual, hour and treatment (as fixed factors) and day (as a random factor), on the transformed mosquito counts. Further post hoc testing of individual variables was performed using a Tukey's Honestly Significant Different test (Tukey's HSD).

## Results

### Study A

In 25 nights, 6,140 mosquitoes were captured comprising 55.6% *Psorophora varipes *(Coquillett) and 24.8% *Aedes ochlerotatus taeniorhynchus*. The average number of mosquito landings on the negative control was 108 per person/hour and there was no significant difference in hourly numbers of landings in this treatment (*F *= 0.896, d.f. = 1, *p *= 0.345).

Each of the four repellents provided excellent protection from host-seeking mosquitoes, and the PMD/LG repellents provided >97% protection up to five hours after application, with T15 and T20 providing 99% protection. DEET (15%) provided 92% (Table 1). GLM analysis showed that there was a significant difference between the four repellents and the negative control, and between DEET and the three PMD based repellents; although there was no significant difference between the three PMD/LG repellents (Tukey's HSD, p < 0.0001) (Table 1). Sources of error in the experimental design were also investigated. There was no significant difference between the collection positions within the field site (*F *= 2.149, d.f. = 4, *p *= 0.76), although individuals varied significantly in their mean attractiveness to mosquitoes/collection ability (*F *= 6.73, d.f. = 4, *p *< 0.0001).

**Table 1:** Efficacy of 4 repellent formulations tested 4 and 5 hours after application during Study A in Guatemala

		Hours post application			
**Treatment**		4	5	Mean	95 % C.I.

**Filler mix control**	AM	115.52	100.48	108.00	55.13 – 96.39
	WM	79.83	66.64	72.74^a^	
	% P	--	--	--	
**C15**	AM	1.28	5.04	3.16	0.46 – 01.38
	WM	0.64	1.40	0.96^b^	
	% P	98.89	94.98	97.07	
**T15**	AM	1.36	1.44	1.40	00.43 – 01.17
	WM	0.79	0.72	0.76^b^	
	% P	98.82	98.57	98.70	
**T20**	AM	0.84	1.84	1.34	0.28 – 00.97
	WM	0.43	0.77	0.59^b^	
	% P	99.27	98.17	98.76	
**15 % DEET**	AM	6.08	11.72	8.90	2.68 – 06.38
	WM	3.63	4.87	4.21^c^	
	% P	94.74	88.34	91.76	

AM = arithmetic mean mosquito landings per person hour.

WM = William's mean mosquito landings per person hour. Means followed by different letters are significantly different.

% P = Percentage protection i.e. 100 - ((mosquito landings on treatment ÷ mosquito landings on control) × 100).

### Study B

In nine nights, 2,358 mosquitoes were captured, of which 86% were *An. darlingi*. The average number of landings on the negative control was 46.28 per person/hour. There was no significant difference in the hourly number of mosquitoes captured from the control (*F *= 1.167, d.f. = 3, *p *= 0.326), or *An. darlingi *(*F *= 1.667, d.f. = 3, *p *= 0.179) which indicates that the repellents' efficacy did not significantly decline during the six hours of the test.

The PMD/LG repellent significantly outperformed DEET, providing an average of 95% protection six hours after application (F = 128.8, d.f. = 2, *p *< 0.0001) (Table 2). In contrast, 20% DEET provided an average of 64% protection over the duration of the trial. Sources of bias were investigated and there was no difference in the number of mosquitoes captured in the three positions within the field (*F *= 0.87, d.f. = 2, *p *= 0.422) or individual variation in attractiveness to mosquitoes/collection ability (*F *= 1.492, d.f. = 2, *p *= 0.230).

**Table 2:** Efficacy of 2 repellents tested 3 to 6 hours after application during study B in Peru.

		All mosquitoes Hours post application						*Anopheles darlingi *Hours post application					
**Treatment**		3	4	5	6	Mean	95 % C.I.	3	4	5	6	Mean	95 % C.I.

**20 % oil control**	**AM**	44.44	62.11	38.44	40.11	46.28	27.97 – 46.58	31.11	54.78	34.11	35.44	38.86	20.05 – 37.64
	**WM**	34.52	53.05	31.46	29.27	36.13^a^		22.10	44.60	23.05	24.79	27.52^a^	
	**% P**	-	-	-	-	-		-	-	-	-	-	
**PMD/LG Repellent**	**AM**	1.33	1.78	1.67	4.78	2.39	0.73 – 2.10	1.11	1.56	1.44	3.89	2.00	0.63 – 01.80
	**WM**	0.82	1.08	1.15	2.52	1.32^b^		0.66	0.93	1.05	2.17	1.14^b^	
	**% P**	97.01	97.13	95.66	88.08	94.84		96.43	97.15	95.78	89.02	94.85	
**20% DEET**	**AM**	15.22	19.78	13.89	18.44	16.83	10.17 – 17.25	13.56	18.33	12.89	17.22	15.50	9.13 – 15.76
	**WM**	10.59	16.12	11.68	15.28	13.28^c^		9.59	14.96	10.59	13.59	12.02^c^	
	**% P**	65.75	68.15	63.86	54.03	63.63		56.41	66.53	62.21	51.41	60.11	

AM = arithmetic mean mosquito landings per person hour.

WM = William's mean mosquito landings per person hour. Means followed by different letters are significantly different.

% P = Percentage protection i.e. 100 - ((mosquito landings on treatment ÷ mosquito landings on control) × 100).

## Discussion and Conclusion

In both field trials, the PMD/LG repellents with fixatives showed excellent efficacy against a broad range of mosquito species, with greater than expected longevity for a 15% PMD formulation. Also, in these trials the PMD/LG repellents showed greater efficacy than corresponding doses of DEET. This is an important point, as several other studies have shown PMD to have longevity similar to [17], or lower than [25], a corresponding dose of DEET. During a 2001 Bolivian field trial against *An. darlingi *where the biting pressure was 75 mosquito landings per person/hour, a repellent containing 30 % PMD in ethanol provided 97 % protection, and 15% DEET provided 85% protection for four hours after application [17]. However, in the current Study A, the repellent containing half that concentration of PMD (T15) provided 99% protection for five hours after application with a biting pressure of 108 mosquitoes per person/hour, compared to 92% for 15% DEET. In Study B, a repellent with 16% PMD provided 95% protection for six hours after application, compared to 64% protection for 20% DEET.

It may be inferred from this that the addition of fixatives to the repellents tested in Guatemala and Peru slowed the release of repellent volatiles, thereby extending the repellents' duration and lowering its cost. Additionally, because PMD is the most costly ingredient in the repellent, the savings realized by a reduction in PMD content from 30% to 15% have reduced the cost of this disease prevention tool even more. There is much evidence that the indigenous poor are less likely to purchase ITNs and repellents, and more likely to rely upon cheaper, less effective methods of personal protection [26]. Therefore, making the repellent available at the lowest cost possible could enhance user acceptance.

The potential benefit of this portable protection to anti-malaria campaigns in the Americas can be seen in the economics of the 1998 malaria epidemic in Peru. It has been estimated that the total cost of treating malaria that year was $190 per individual case [27]. That includes all associated state health expenses and the costs to the family for treatment, lost income, and death. Adjusted for inflation, that is approximately $250 in 2007. However, the estimated annual cost per person for the repellent intervention (coverage during the 7-month transmission season at $0.024/day) is $5.00. That is 2% of the estimated total cost of treatment today. Consequently, the PMD/LG repellent may be an excellent candidate for incorporation into existing vector control strategies.

If such economies could be achieved in Peru, while reducing the disease burden from malaria, this model might be applicable to other regions where early evening biting is problematic. Essentially, the model for "repelling" malaria proposes the following: where the crepuscular feeding behavior of the most significant malaria vectors is already established, or where it may be shifting to early evening biting or outdoor resting [28] (from selection due to IRS and/or ITN use), it may be possible to achieve a reduction of 60% in malaria cases [13]. This could be achieved by saturation of at-risk communities (to avoid diversion of malaria vectors to non-users) with high-efficacy LCRs that are affordable and aromatically attractive to the indigenous poor. Combined with ITNs, the reduction could be substantially greater.

A five-month Phase 3 study, currently under way in the Peruvian Amazon, is providing an opportunity to measure the parameters of this model on 16 population clusters. With 3,300 subjects divided into three cohorts (Repellent Only, Repellent + ITNs, and No Intervention), this community-wide study marks the first time that the effect of a Repellent Only intervention on malaria rates has been measured in a discrete population group in the tropics. If the LCR's demonstrated capacity to repel malaria vectors leads to a measurably significant reduction in the infection rate, the Puerta del Cielo Foundation will begin distributing repellents and treated bednets at cost to poor malaria-endemic communities throughout the Peruvian Amazon.

## Competing interests

The research that appears in this manuscript was funded and directed by S.T. Darling with organizational support from Puerta del Cielo, a Guatemalan non-profit foundation. A patent in Mr. Darling's name is pending on one of the repellent formulations tested in these trials. When approved, this patent will be conveyed to an NGO that will distribute the repellent at cost (not-for-profit) to malaria-endemic communities in the Americas and elsewhere. Mr. Darling will not profit from its development or sale. Under Mr. Darling's direction, Sarah Moore was paid to carry out specific tasks related to his repellent research. The remaining co-authors gave their services freely to this effort and, therefore, retained their independence from the funder.

## Authors' contributions

SJM designed the study protocols, supervised the repellent trials, collected and analysed the data, and co-authored the manuscript. STD directed the repellent research, designed the repellent, co-authored the manuscript, and was responsible for editing. MS: provided translation and logistical support in Iquitos, Peru. NP provided translation and organizational help through CDC/MERTU and Universidad del Valle in Guatemala. GJD implemented the study protocols in Peru, guided them through ethics committees there, and contributed to editing the manuscript.
